# Premature Discontinuation of Prospective Clinical Studies Approved by a Research Ethics Committee – A Comparison of Randomised and Non-Randomised Studies

**DOI:** 10.1371/journal.pone.0165605

**Published:** 2016-10-28

**Authors:** Anette Blümle, Stefan Schandelmaier, Patrick Oeller, Benjamin Kasenda, Matthias Briel, Erik von Elm, on behalf of the DISCO study group

**Affiliations:** 1 Cochrane Germany, Medical Center-University of Freiburg, Faculty of Medicine, University of Freiburg, Freiburg, Germany; 2 Basel Institute for Clinical Epidemiology and Biostatistics, University Hospital Basel, Basel, Switzerland; 3 Department of Clinical Epidemiology and Biostatistics, McMaster University, Hamilton, Ontario, Canada; 4 Cochrane Switzerland, Institute of Social and Preventive Medicine (IUMSP), Lausanne University Hospital, Lausanne, Switzerland; BRAC, BANGLADESH

## Abstract

**Background:**

Premature discontinuation of clinical studies affects about 25% of randomised controlled trials (RCTs) which raises concerns about waste of scarce resources for research. The risk of discontinuation of non-randomised prospective studies (NPSs) is yet unclear.

**Objectives:**

To compare the proportion of discontinued studies between NPSs and RCTs that received ethical approval.

**Methods:**

We systematically surveyed prospective longitudinal clinical studies that were approved by a single REC in Freiburg, Germany between 2000 and 2002. We collected study characteristics, identified subsequent publications, and surveyed investigators to elucidate whether a study was discontinued and, if so, why.

**Results:**

Of 917 approved studies, 547 were prospective longitudinal studies (306 RCTs and 241 NPSs). NPSs were on average smaller than RCTs, more frequently single centre and pilot studies, and less frequently funded by industry. NPSs were less frequently discontinued than RCTs: 32/221 (14%) versus 78/288 (27%, p<0.001, missing data excluded). Poor recruitment was the most frequent reason for discontinuation in both NPSs (36%) and RCTs (37%).

**Conclusions:**

Compared to RCTs, NPSs were at lower risk for discontinuation. Measures to reliably predict, sustain, and stimulate recruitment could prevent discontinuation of many RCTs but also of some NPSs.

## Introduction

In recent years, many methodological investigations and guidance documents about randomised controlled trials (RCTs) have been published, whereas problems in the planning and conduct of other prospective clinical studies such as non-randomised controlled trials, single arm trials or cohort studies (non-randomised prospective studies, NPSs) are less well known.

A major reason for failure of RCTs is premature discontinuation; about one quarter of planned RCTs are prematurely discontinued, mostly due to recruitment problems [1–5]. Discontinuation is rarely reported to research ethics committees (RECs) and about half of discontinued RCTs remain unpublished [1, 3]. This is a serious ethical concern for volunteering participants and the society at large as it represents a waste of scarce resources, a loss of valuable research data, and missed opportunities to learn from failure [1, 2, 6].

Similar ethical implications would apply to NPSs. However, their premature discontinuation has not been investigated in depth so far. (Retrospective or cross-sectional studies are not discussed in this paper because the mechanisms for discontinuation differ from prospective studies. For instance, they cannot be discontinued for slow benefit, harm, futility, or slow recruitment). It is unknown whether the risk and reasons for discontinuation of NPSs differ from RCTs. Pilot or feasibility NPSs conducted on selected populations may be less prone to discontinuation due to poor recruitment than confirmative RCTs that need to achieve a certain sample size to establish the effectiveness of a treatment. A study on a sample of cardiovascular trials registered in ClinicalTrials.gov showed that besides funding by federal agencies and behavioural therapies, a single arm study design is associated with a lower risk of early termination due to poor recruitment [7]. Furthermore, studies suggest that it is easier to recruit participants into NPSs [8, 9]. On the other hand, NPSs may be more frequently explorative in nature including first-in-human studies or early pharmacological studies [7]. Those studies are typically surrounded by more uncertainties concerning the potential benefit and harms an intervention could have for patients, and may thus bear a higher risk for discontinuation.

The aim of this analysis was to study the discontinuation of clinical studies that were approved by a REC, and to compare the prevalence of and reasons for discontinuation between NPSs and RCTs.

## Methods

We had access to all study protocols submitted to the REC of the University of Freiburg / Germany from 2000 to 2002, including RCTs and NPS [10, 11]. If a study protocol described two or more studies, we considered each study separately.

**Fig 1:**
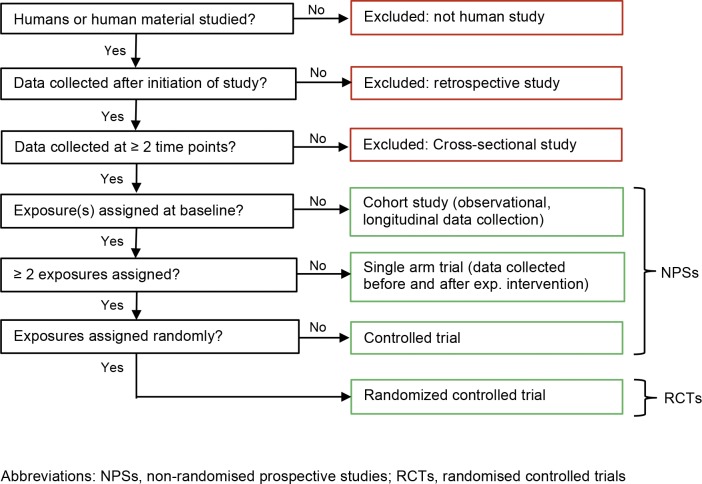
Algorithm for classifying study designs.

We included studies that 1) enrolled patients or healthy volunteers (hereafter referred to as ‘participants’), 2) collected baseline data after initiation of the study (prospective studies), and 3) had at least one follow-up time point regardless of time elapsed since baseline (longitudinal studies). We excluded retrospective longitudinal and cross-sectional studies because our main outcome “discontinuation of recruitment and/or follow-up” would not apply. Furthermore, we excluded studies if we certainly knew that they were never started or were still on-going at time of data collection (Fig 1). We considered a study on-going if investigators indicated this in response to our survey and if results had not been published.

We used the following definitions to classify NPSs (Fig 1):

1. Controlled trial: Participants are systematically assigned to two or more parallel exposure (intervention) group defining the study arms. An exposure (intervention) could be in one time point or repeated / continued over a defined period of time.
2. Single arm trial: All participants are assigned to one exposure (intervention) group.
3. Cohort study: Participants are not actively assigned to exposure groups; those are defined by observed characteristics or exposures.

Details of data collection have been described previously [10, 11]. REC files included local application forms, the study protocols with amendments and correspondence with the REC. We extracted information about study centre status (multicentre international/national, single centre), sample size, pilot study, medical field, inclusion criteria, and source of funding (industry/non-industry). We considered a study industry-sponsored if the protocol clearly named the sponsor, displayed a company or institution logo prominently, mentioned affiliations of protocol authors, or included statements about data ownership or publication rights, or statements about full funding by industry or public funding agencies. If only some study material (e.g. the experimental drug) was provided by a private company and academic investigators wrote the study protocol, we did not consider the study industry-sponsored.

We used pre-piloted standardized forms for all extracted information and provided detailed written data extraction instructions. To minimize extraction errors, we conducted formal calibration exercises with all data extractors and extracted 30% of the RCTs in duplicate. NPSs data were extracted by one investigator (PO). All database entries were checked for plausibility and about one third of the data was cross-checked by a second investigator (AB), so that we are confident that we achieved a high level of accuracy. If the investigator in charge could not decide on how to extract data (e.g. when classifying studies by design), the issue was discussed with a second investigator. If data extraction still remained unclear, a third investigator (SS) was involved to reach consensus.

In a second step, we established a specific search strategy for each study protocol using relevant keywords from the protocol such as experimental drug, study name or acronym, studied disease or condition or names of applicants. We searched the databases Medline, Web of Science, publication registry of the University of Freiburg, via Medpilot the databases of Current Contents Medizin, the publishers Hogrefe, Karger, Kluwer, Springer and Thieme, and Google Scholar. For RCTs we also searched the Cochrane Central Register of Controlled Trials (CENTRAL) of the Cochrane Library. When we calculated the proportion of published studies, we considered only articles published in peer-reviewed journals; in contrast to conference abstracts and grey literature, peer reviewed journal articles are indexed in electronic databases and can be identified reliably. Estimates of publication proportions based on peer-reviewed articles are therefore more reliable but also more relevant. Literature searches were conducted in 2011/2012 for study protocols of the year 2000 and in 2009/2010 for those of the years 2001 and 2002, and in 2014 for all RCTs.

We contacted the applicants of all included protocols by a personalised letter including a questionnaire to confirm the publications identified by us and to ask for additional publications (S1 Appendix). We also asked about premature discontinuation and the reason(s) thereof. The survey of investigators was conducted in 2007 for protocols of the year 2000, in 2010 for protocols of the years 2001 and 2002, and in 2013 for all RCTs. The survey response rate in Freiburg was 90.0% for RCTs and non-RCTs. We grouped the reasons for study discontinuation provided by the investigators into the following categories: poor recruitment, benefit, harm, futility, lack of funding or other reasons (including administrative reasons such as retirement or change of institute of principal investigator or disagreement with sponsor).

Our primary analysis was to compare the proportion of discontinued studies between NPSs and RCTs. We calculated proportions based on complete cases (excluding studies with missing status information) and conducted sensitivity analyses assuming that a) unclear/missing studies were completed, b) unclear/missing studies were discontinued, or c) half of unclear/missing studies were discontinued. The latter assumption that unclear/missing studies were more likely to be discontinued was based on the observation that unknown status was associated with non-publication which is known to be associated with discontinuation [1]. In another sensitivity analysis, we excluded studies stopped for harm, benefit, or futility. The rationale was to enable a ‘fairer’ comparison because cohort studies cannot be stopped for these reasons. We used the chi-square test to test for differences in proportions (based on the assumption that studies approved in Freiburg are a random sample of studies approved in comparable jurisdictions and future studies) and a type I error of 5% as threshold for statistical significance.

To explore potential differences across countries and jurisdictions, we compared the study characteristics and discontinuation between the RCTs approved in Freiburg and RCTs approved in Canada (Hamilton) and Switzerland (Basel, Lausanne, Zurich, and Lucerne) at the same time period. Details about this cohort of 1017 RCTs were reported earlier [1, 12].

## Results

### Included studies

**Fig 2:**
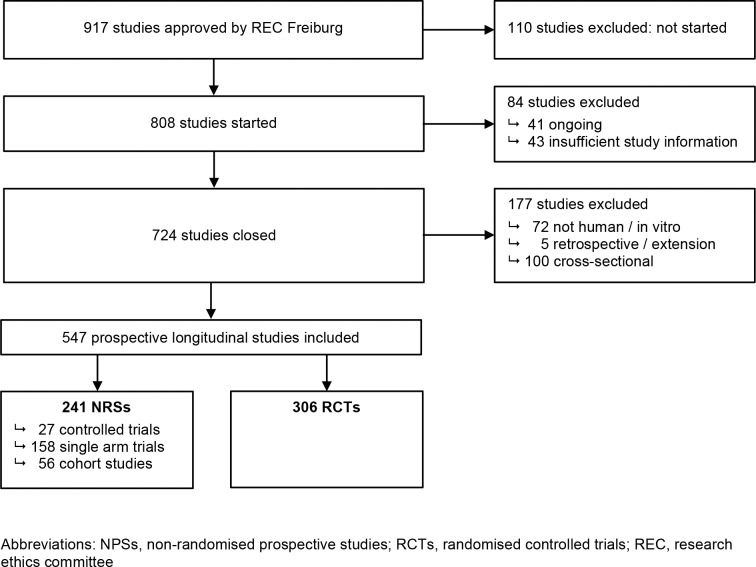
Study selection.

We identified 917 studies that were approved by the REC in Freiburg (Fig 2). After excluding studies that were never started, still on-going, in vitro or other non-human studies, or of cross-sectional or retrospective design, our final data set for analysis comprised 547 prospective longitudinal studies. Of those, 306 were RCTs and 241 were NPSs (27 controlled trials, 158 single arm trials, and 56 cohort studies).

### Study characteristics

Most NPSs (92%) and RCTs (92%) were conducted in adults. NPSs were on average smaller than RCTs, more frequently single centre and pilot studies, and less frequently industry-sponsored (Table 1). Most NPSs and RCTs were in oncology (20%; 22%) and neurology (11%; 9%). NPSs were less often conducted in cardiovascular medicine (3%; 9%) and more often in dental medicine (6%; 2%). Fourty-nine precent of NPSs were published compared to 59% of RCTs.

**Table 1:** Study characteristics.

Study characteristics	REC Freiburg					Other RECs^1^
	Controlled trial	Single arm trial	Cohort study	Total NPSs	RCTs	RCTs
**Total n**	27 (100)	158 (100)	56 (100)	241 (100)	306 (100)	711 (100)
Sample size provided	27 (100)	154 (97)	54 (96)	235 (98)	302 (99)	697 (98)
Median; IQR	30; 20–48	60; 30–238	115;50–300	60; 30–230	200; 81–449	240; 60–600
**Centre status**						
Single centre	25 (93)	46 (29)	37 (66)	108 (45)	70 (23)	191 (27)
Multi centre—national	1 (4)	45 (29)	13 (23)	59 (25)	67 (22)	92 (13)
Multi centre—international	1 (4)	66 (42)	6 (11)	73 (30)	155 (51)	404 (57)
Unclear/missing	0	1 (1)	0	1 (<1)	14 (5)	24 (3)
**Labelled as pilot study**	14 (52)	34 (22)	19 (34)	67 (28)	36 (12)	47 (7)
**Sponsorship**						
Industry^2^	9 (33)	99 (63)	10 (18)	118 (49)	182 (59)	455 (64)
Non-industry	18 (67)	59 (37)	46 (82)	123 (51)	124 (41)	256 (36)
**Medical field**						
Oncology	3 (11)	40 (25)	7 (12)	50 (21)	66 (22)	90 (13)
Cardiovascular	0 (0)	5 (3)	3 (5)	8 (3)	27 (9)	90 (13)
Infectious disease	2 (7)	12 (8)	5 (9)	19 (8)	23 (8)	78 (11)
Endocrinology	1 (4)	3 (2)	1 (2)	5 (2)	14 (5)	64 (9)
Neurology	3 (11)	18 (11)	8 (14)	29 (12)	27 (9)	39 (5)
Gastroenterology	0	1 (1)	1 (2)	2 (1)	12 (4)	52 (7)
Respiratory	0	4 (3)	3 (5)	7 (2)	15 (5)	46 (6)
Psychiatry	3 (11)	15 (9)	4 (7)	22 (9)	20 (7)	25 (4)
Dermatology	4 (15)	8 (5)	2 (4)	14 (6)	21 (7)	16 (2)
Haematology	0	9 (6)	2 (4)	11 (5)	9 (3)	27 (4)
Dental / facial surgery	7 (16)	6 (4)	1 (2)	14 (6)	5 (2)	1 (0)
Other	4 (15)	37 (23)	19 (34)	60 (25)	67 (22)	183 (26)
**Age group**						
Adult	26 (96)	148 (94)	47 (84)	221 (92)	283 (92)	648 (91)
Paediatric	1 (4)	10 (6)	9 (16)	20 (8)	23 (8)	63 (9)
**Participants**						
Patients	18 (67)	142 (90)	45 (80)	205 (85)	282 (92)	612 (86)
Healthy volunteers	8 (30)	11 (7)	3 (5)	22 (9)	24 (8)	99 (14)
Both	1 (4)	5 (3)	8 (14)	14 (6)	0	0
**Published in peer-reviewed journal**	15 (56)	74 (47)	29 (52)	118 (49)	180 (59)	387 (54)

^1^ approved by 5 research ethics committees in Switzerland and Canada [1].

^2^ includes 37 RCTs and 13 non-RCTs with industry funding but no industry involvement, i. e. in study planning, management or analysis of data.

All percentages (in brackets) refer to the total number of the respective column

Abbreviations: NPSs, non-randomised prospective studies; RCTs, randomised controlled trials; IQR, interquartile range.

### Study discontinuation

Overall, NPSs were less frequently discontinued than RCTs (14% versus 27%, missing excluded, p<0.001) (Tables 2 and 3). Sensitivity analyses using different assumption for missing data only slightly changed these proportions and the differences between NPSs and RCTs. In the sensitivity analysis excluding studies stopped for harm, benefit, or futility the difference between NPSs and RCTs was no longer statistically significant (p = 0.057, missing excluded, see Table 3). Poor recruitment was the most frequent reason for discontinuation in both NPSs (37%) and RCTs (36%) (Table 2). Completion status was very similar in RCTs approved in Freiburg and in Canada or Switzerland.

**Table 2:** Study status, reasons for discontinuation.

Study characteristics	REC Freiburg					Other RECs^1^
	Controlled trial	Single arm trial	Cohort study	Total NPSs	RCTs	RCTs
**Total n**	27	158	56	241	306	711
**Study status**						
Completed^2^	23	124	42	189	210	474
Unclear/missing^2^	2	10	8	20	18	62
Discontinued^2^	2	24	6	32	78	175
**Reasons for discontinuation**						
Poor recruitment	1 (50%)	6 (27%)	4 (67%)	11 (37%)	28 (36%)	73 (42%)
Withdrawal by sponsor/ lack of funding	0	5 (23%)	0	5 (17%)	3 (4%)	2 (1%)
Benefit	0	0	0	0	6 (8%)	3 (2%)
Harm	1 (50%)	2 (95%)	0	3 (10%)	7 (9%)	17 (10%)
Futility	0	3 (14%)	0	3 (10%)	16 (21%)	21 (12%)
Other reasons^3^	0	6 (27%)	2 (33%)	8 (27%)	17 (22%)	35 (20%)
Unclear/missing	0	2	0	2	1	24

^1^ approved by 5 research ethics committees in Switzerland and Canada [1].

The numbers in brackets are proportions (column %) based on complete cases (excluding unclear/missing)

^2^ Please see Table 3 for proportions

^3^ Other reasons: administrative; retirement; change of institute of applicant; lack of staff resources; lack of flexibility of the system; logistical problems, i.e. interdisciplinary study organisation and logistics; number of participants too small; studies conducted by other researcher were larger and more meaningful; pilot phase failed, test material was insufficient; study data were deleted during maintenance works by the technician; change of study design; change of topic; termination of the liver transplant programme at the University Medical Center Freiburg by the government; shifting of the research focus of the study investigator

Abbreviations: NPSs, non-randomised prospective studies; RCTs, randomised controlled trials.

**Table 3:** Proportion of discontinued trials.

	REC Freiburg						Other RECs^1^
	Controlled trial	Single arm trial	Cohort study	Total NPSs	RCTs	Comparison total NPS vs RCTs P	RCTs
**All studies**							
Unclear/missing = excluded	8%	16%	13%	14%	27%	<0.001	27%
Unclear/missing = completed	7%	15%	11%	13%	25%	<0.001	25%
Unclear/missing = discontinued	15%	22%	25%	22%	31%	0.014	33%
Unclear/missing = 50% completed, 50% discontinued	11%	18%	18%	17%	28%	0.004	29%
**Excluding studies stopped for harm, benefit, futility**							
Unclear/missing = excluded	4%	13%	13%	12%	19%	0.057	22%
Unclear/missing = completed	4%	12%	11%	11%	18%	0.047	20%
Unclear/missing = discontinued	12%	19%	25%	20%	24%	0.251	29%
Unclear/missing = 50% completed, 50% discontinued	8%	16%	18%	15%	21%	0.128	25%

^1^ approved by 5 research ethics committees in Switzerland and Canada [1].

Estimated proportion of discontinued studies applying different assumptions for missing data with or without excluding studies that were stopped for intervention-related reasons such as harm, benefit, and futility (see text for a rationale).

Abbreviations: NPSs, non-randomised prospective studies; RCTs, randomised controlled trials.

## Discussion

We compared the risk of discontinuation between NPSs and RCTs in a sample of clinical studies approved by a German REC.

Overall, NPSs were at lower risk of discontinuation than RCTs (14% versus 27%, p<0.001). The difference was robust to sensitivity analyses using different assumptions for missing data, however, when we excluded studies stopped for harm, benefit, or futility, the difference in the proportion of discontinued studies diminished and was no longer significant. This is obviously due to the fact that cohort studies are not being stopped for harm, benefit, or futility.

A strength of our study is that we had access to the full REC correspondence from a period of three years. Consequently, we could take into account relevant information from the application form, the study protocol with its amendments, patient information sheets and additional correspondence. In addition, we achieved a high response rate in our survey. The included RCTs were remarkably similar to RCTs that were approved at the same time by five RECs in Switzerland and Canada. This increases our confidence that the findings will be generalizable to clinical research settings in other high-income countries.

A main limitation of our study was that for some studies only little information was available, e.g. due to missing or poor reporting in study documents. When a study protocol was missing, we extracted relevant information from application forms and patient information sheets. While all RCTs conducted in the jurisdiction of the REC needed to obtain ethical approval, the regulations were less strict for NPSs between the years 2000 and 2002. We could not quantify the number of NPSs that were never assessed by the REC. Consequently, it is possible that our sample of NPSs overrepresented more challenging studies for which ethical approval was deemed critical and that it might not be representative of NPSs in general. Finally, when we applied statistical tests and formulated our conclusions, we implicitly regarded the studies approved in Freiburg as being representative of other clinical studies approved in other jurisdictions. This assumption is supported by the fact that characteristics of RCT in Freiburg were very similar to characteristics of RCTs in Switzerland and Canada (Table 1). However, results may differ among studies performed in jurisdictions where unique challenges exist, such as developing countries.

Only few previous studies have reported on the risk of discontinuation in NPSs. We screened two systematic reviews including methodological studies on studies approved by RECs or included in trial registries [13, 14]. One study followed a cohort of 367 studies (137 RCTs and 230 studies of other designs) approved by a single REC in Oxford, UK, between 1984 and 1987 [15]. It did not investigate differences between study designs due to the low number of discontinued studies. Similar to our study, poor recruitment was the main reason for study discontinuation across designs. Compared to completed studies, discontinued studies were more likely to be non-comparative (33% vs 27%) and single centre (83% vs 76%). Another study was based on cardiovascular studies registered in ClinicalTrials.gov [7]. Again, slow recruitment was the main reason for study discontinuation. In addition, single arm design was independently associated with lower risk for recruitment failure. Our sample did not include enough single arm trials to explore this potential association.

## Conclusions

Our analysis suggests that NPSs may be at lower risk for discontinuation than RCTs. Poor recruitment was the main reason for study discontinuation in both RCTs and NPSs. Measures to reliably predict, stimulate and sustain recruitment performance may prevent the discontinuation of many NPSs and RCTs in the future [16–18].

## Supporting Information

S1 Appendix Standardized Survey Questionnaires. The anonymized data set is accessible on https://freidok.uni-freiburg.de/data/11187 (10.6094/UNIFR/11187).
(PDF)
