# Supplementary material for: Premature Discontinuation of Prospective Clinical Studies Approved by a Research Ethics Committee – A Comparison of Randomised and Non-Randomised Studies
Source: PLoS One. 2016 Oct 28;11(10):e0165605. doi: 10.1371/journal.pone.0165605 (PMC5085068; doi:10.1371/journal.pone.0165605)
Supplement: S1 Appendix — The anonymized data set is accessible on https://freidok.uni-freiburg.de/data/11187 (10.6094/UNIFR/11187). (PDF) [file pone.0165605.s001.pdf]

S1 Appendix

Standardized Survey Questionnaire (RECs Switzerland and Canada)

Trial status

1. WAS THE TRIAL STARTED AT ALL?

   YES ▢ **NO** ▢ **UNKNOWN** ▢

   - If NO, check the appropriate box (← applies only to **multicenter** studies):
     - *Whole study* was not started ▢
     - Not started only in *your center* ▢

2. IS THE TRIAL STILL RUNNING?

   YES ▢ **NO** ▢ **UNKNOWN** ▢

   - IF **YES** (Check all that apply):
     - Recruitment not completed yet ▢
     - Recruitment completed ▢
     - Data collection completed ▢

If the **trial is <u>still running</u>**, the **questionnaire** is **completed** for you.

If the **trial <u>is NOT running anymore</u>**, **please proceed** with this questionnaire.

3. HAS THE TRIAL BEEN DISCONTINUED PREMATURELY?

   YES ▢ **NO** ▢ **UNKNOWN** ▢

   - If **YES** (Check all that apply):
   - Stopped because of slow recruitment ▢
   - Stopped for harm ▢
   - Stopped for benefit ▢
   - Stopped because of evidence from other trials ▢
   - Stopped for futility ▢
   - Other reason (please specify) ▢

Specification/comments:____

Publication status of the trial

4. HAVE THE RESULTS OF THIS TRIAL BEEN PUBLISHED?

YES ▢ **NO** ▢ **UNKNOWN** ▢

If **YES,** what was the type of the publication (CHECK ALL THAT APPLY)

- Publication in **Journal** ▢
- **Abstract** presentation at conference ▢
- **Internet Report** ▢
- **Other form** (please specify below) ▢

PLEASE GIVE **ALL REFERENCES** OF THE PUBLICATION WITH **NAME OF THE FIRST AUTHOR, TITLE OF THE PUBLICATION, NAME OF THE JOURNAL/ CONFERENCE/OTHER FORM** AND **PUBLICATION YEAR**. FOR **INTERNET PUBLICATIONS** PLEASE PROVIDE THE WEBADRESS**:**

____

____

____

____

Standardized Survey Questionnaire (REC Freiburg, Germany)

Antragsteller:

Titel des Projekts:

Antrag Nummer:

Aktueller Stand der Studie:

Diese Studie

☐ wurde gemäß Protokoll abgeschlossen

☐ wurde vorzeitig beendet. Grund: ....

☐ wurde nicht begonnen: ☐ als ganze Studie

☐ nur in Ihrem Zentrum

☐ läuft noch: ☐ Rekrutierung abgeschlossen

☐ Datensammlung abgeschlossen

☐ vorläufig publiziert (bitte Referenz unten angeben)

Bitte bestätigen Sie für jede der folgenden Publikationen, ob sie dem oben genannten Projekt zuzuordnen ist:

1.) ………

2.) ………..

Bitte geben Sie die Referenzen (Autoren, Titel, Zeitschrift, Band, Seitenzahl) weiterer Publikationen an. Bitte evtl. Zusatzblatt benutzen.

Originalarbeiten:

1.: ....

2.: .... etc.

2. Abstracts und Buchbeiträge:

1.: ....

2.: .... etc.
